# Morphological and Histological Variations of the Reproductive Organs During the Annual Cycle in a Neotropical Bat: Peters' Ghost‐Faced Bat *Mormoops megalophylla* (Chiroptera: Mormoopidae)

**DOI:** 10.1002/jmor.70066

**Published:** 2025-07-15

**Authors:** Gihovani Ademir Samano‐Barbosa, Ixchel Rojas‐Martínez, Sergio Leonardo Porto‐Ramírez, Fernando Salgado‐Mejia, Ahiezer Rodríguez‐Tobón, Arturo Salame‐Méndez, Edith Arenas‐Ríos, Luis Manuel Guevara‐Chumacero, Ricardo López‐Wilchis

**Affiliations:** ^1^ Doctorado en Ciencias Biológicas y de la Salud Universidad Autónoma Metropolitana Ciudad de México Mexico; ^2^ Laboratorio de Biología y Ecología de Mamíferos, Departamento de Biología Universidad Autónoma Metropolitana‐Iztapalapa Ciudad de México Mexico; ^3^ Posdoctorado por México CONAHCYT Universidad Autónoma Metropolitana‐Iztapalapa Ciudad de México Mexico; ^4^ Laboratorio de Ecofisiología y Cambio climático, Departamento de Biología de la Reproducción Universidad Autónoma Metropolitana‐Iztapalapa Ciudad de México Mexico; ^5^ Laboratorio de Morfofisiología y Bioquímica del Espermatozoide, Departamento de Biología de la Reproducción Universidad Autónoma Metropolitana‐Iztapalapa Ciudad de México Mexico

**Keywords:** biology, folliculogenesis, reproduction, sinistral dominance, spermatogenesis

## Abstract

Recent studies have emphasized the ecological significance of bats as insect regulators. This recognition has prompted an increased scientific interest in *Mormoops megalophylla*, a notable neotropical insectivorous bat species. The extant literature on its biology remains limited and substantial knowledge gaps persist, particularly regarding its reproductive cycle. This study sought to examine morphological and histological variations in the reproductive organs of male and female *M. megalophylla* over the annual cycle to elucidate the stages of its reproductive process. Over the course of the year, five sexually mature individuals of each sex were sampled monthly, culminating in a total of 120 specimens, to document variations in external sexual characters, the testes, epididymides, uterus, and ovaries; all sampled individuals underwent morphological, morphometric, and histological analyses. The findings indicate that *M. megalophylla* has migratory testes with seasonal spermatogenesis occurring from October to December, a bicornuate uterus, and a single folliculogenic period that is synchronized with spermatogenesis. This suggests a monoestrous, seasonal, and synchronous reproductive pattern. At the population level, copulation occurs between November and December, gestation occurs between December and May, and parturition occurs between late May and early June. The lactation period extended from June to September.

## Graphical abstract

*Mormoops megalophylla* exhibits a seasonal monoestrous reproductive pattern. Spermatogenesis and folliculogenesis occurs from October to December, mating takes place in November–December, gestation lasts from December to May, and lactation extends from June to September. Birth and lactation coincide with the rainy season, aligning with the reproductive strategies of Neotropical bats.

## 1 Introduction

Recent research highlights the ecological importance of bats, especially their essential role in controlling insect populations (Castillo‐Figueroa 2020; Maslo et al. 2022; Ramírez‐Fráncel et al. 2022; Tuneu‐Corral et al. 2023; Prakarsa et al. 2024; Yu and Muchhala 2024). This has fueled increasing scientific interest in insectivorous bats, particularly those in the Neotropics.

The Neotropics host approximately 415 bat species (Wilson and Mittermeier 2019; Simmons and Cirranello 2024), a highly diverse group with unique reproductive strategies that distinguish them from other mammals. These strategies, ranging from monoestrous cycles, characterized by a single period of reproductive activity per year, to polyestrous cycles, in which multiple reproductive events can occur annually, may be seasonally restricted or occur year‐round, with considerable variation across and within species. These patterns influence critical reproductive stages, including gametogenesis, mating, gestation, parturition, and lactation (Krutzsch 2000; Racey and Entwistle 2000). However, data on their reproductive biology remain limited.

Understanding the reproductive patterns of insectivorous bats and their temporal and spatial variations is vital for conservation efforts and the preservation of ecosystem services.

Peters' ghost‐faced bat (*Mormoops megalophylla* Peters, 1864), a member of the Mormoopidae, exemplifies these knowledge gaps. Mormoopids, a group of 13 species in two genera, inhabit the Neotropics and are specialized insectivores with overlapping geographical distributions.

*M. megalophylla* is a medium‐sized bat with forearms measuring 49–61 mm. It resides in warm caves (28°C–40°C) with high humidity (> 90%) and ranges from sea level to 3000 m across diverse biomes, including tropical forests, savannas, and shrublands. Its distribution extends from the southern United States through Mexico to northern South America and the Caribbean (Rezsutek and Cameron 1993; Pavan et al. 2019).

This species plays a crucial ecological role in the regulation of insect populations, including agricultural pests. Understanding how environmental factors affect reproductive success is critical, particularly because habitat loss and climate change pose increasing threats. Variations in reproduction could undermine the long‐term survival of populations and their ecological roles.

Despite their broad distribution and importance, the reproductive biology of the Mormoopidae remains poorly understood. For *M. megalophylla*, the literature often provides fragmented or contradictory findings, complicating efforts to clarify its reproductive strategy. While a seasonal monoestrous pattern has been suggested, most studies rely on external morphological data, as seen in the works by Torres‐Flores et al. (2012) and Hernández‐Aguilar and Santos‐Moreno (2020). In addition, published records have shown considerable differences in the timing of the reproductive events. However, we believe that the reported variations, at least in Mexico, could be due to methodological differences, such as limited sampling throughout the annual cycle, a lack of histological validation of reproductive stages, or a sole reliance on external morphological characters. These limitations highlight the need to accurately determine the reproductive cycle of this species. This study aimed to address these shortcomings by providing a comprehensive characterization of the species' reproductive processes by analyzing morphological and/or histological changes in the testes, epididymides, uterus, and ovaries throughout an annual cycle.

## 2 Materials and Methods

### 2.1 Study Area

**Figure 1:**
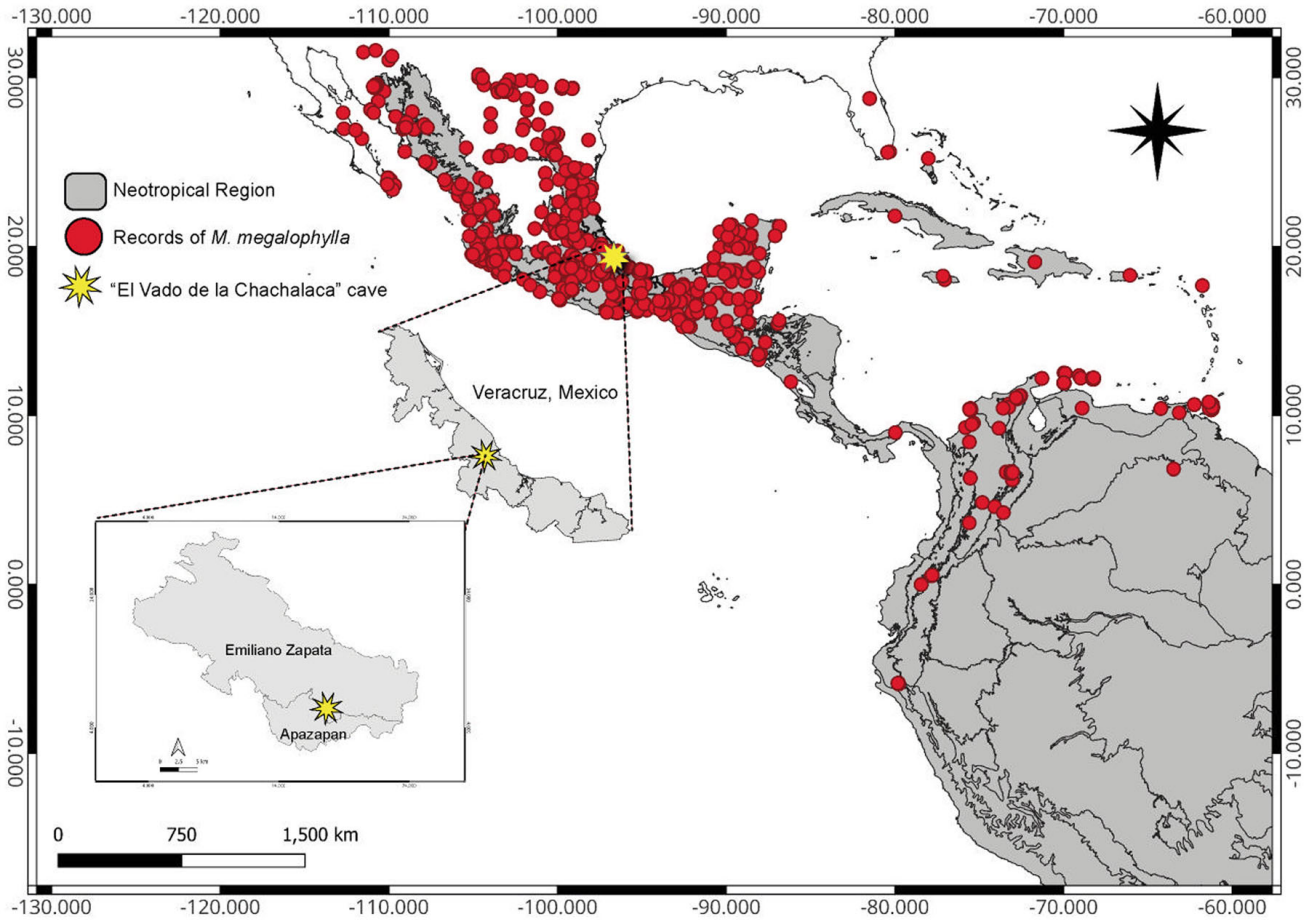
Map illustrating the research site (denoted by a yellow star) and documented occurrences of *Mormoops megalophylla* (represented by red dots) within the neotropical region (shaded in gray).

**Figure 2:**
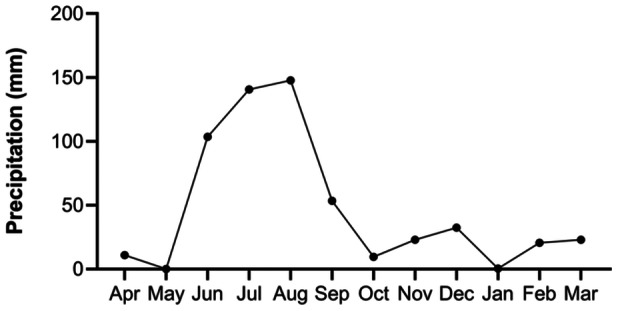
Precipitation levels (mm) recorded in the study area between April 2016 and March 2017.

The study area was located in the central zone of the geographic distribution of species within the Neotropical region (Figure 1). The study took place in “El Vado de la Chachalaca” cave (19° 21′ 12.09″; N; −96° 39′ 30.27″ W, 449 masl), located near Villa de Emiliano Zapata, Veracruz, Mexico. The area's original vegetation primarily consists of tropical deciduous forest (Rzedowski 2006), but much of it has since been replaced by secondary forest fragments, remnants of tropical deciduous forest, and land repurposed for agriculture and livestock. The region experiences an average annual temperature of 25.2°C, with rainfall peaking during summer and early fall (June–September), followed by an extended dry period from October to May (Donner and Arana 2011). Precipitation data were obtained from the “El Carrizal” meteorological station (code 30021), during the period from April 2016 to March 2017, located in the municipality of Emiliano Zapata, Veracruz, and operated by the Sistema Meteorológico Nacional de México (Figure 2).

### 2.2 Biological Sampling

Sampling was conducted monthly from April 2016 to March 2017. Harp traps were positioned at the cave entrance, and the capture period continued until 5 adult males and 5 adult females of *M. megalophylla* (Peters, 1864) were collected each month, resulting in a total sample size of 60 males and 60 females (120 individuals). Adult specimens were selected based on the complete closure of the metacarpophalangeal epiphysis of the fourth finger (Kunz and Anthony 1982; Anthony 1988), and their reproductive conditions were determined according to the external characteristics described by Entwistle et al. (1998) and Sedgeley et al. (2012). Bat species identification was performed using specialized identification keys (Medellín et al. 2008; Álvarez‐Castañeda et al. 2017).

Standard morphometric measurements were performed for each bat specimen. Forearm length was measured using calipers (±0.1 mm), whereas total tail, leg, and ear lengths were determined using a scale ruler. Body mass was measured using an electronic balance (SCALE, 100 g/0.01 g). Following morphometric measurements, the bat was euthanized via cervical dislocation, the reproductive tract was dissected, and the testes, epididymides, and uterus were examined in situ, following the protocols of Gustafson (1979) and Danmaigoro et al. (2014).

The pelvic region was excised and photographed using a Canon 2000D digital camera with an EF100 mm f/2.8 L MACRO IS USM lens. Each pelvic sample was preserved in 10% formalin buffered with monobasic and dibasic sodium phosphate (Montalvo‐Arenas 2010).

The capture, handling, and euthanasia procedures followed the guidelines established by Wilson et al. (1996) and Sikes and The Animal Care Use Committee of the American Society of Mammalogists (2016). The research protocol was approved by the Ethics Committee of the Division of Biological and Health Sciences, Universidad Autónoma Metropolitana‐Iztapalapa (Anónimo 2010). Specimens were collected under permit SGPA/DGVS/003061/18 from the Ministry of Environment and Natural Resources (SEMARNAT).

*M. megalophylla* is not classified under any threat category in Mexico's NOM‐059‐SEMARNAT, 2010 for the protection of native wildlife species (SEMARNAT 2010). According to IUCN, it is categorized as a species of “Least Concern,” although its population is currently experiencing a decline (Dávalos et al. 2019).

### 2.3 Laboratory Work

#### 2.3.1 Morphometric Analysis

Body condition index (BCI) was calculated using the formula weight/forearm length. This index provides an indirect measure of body condition and energy reserves, useful for evaluating health status during the reproductive cycle (Speakman and Racey 1986).

**Figure 3:**
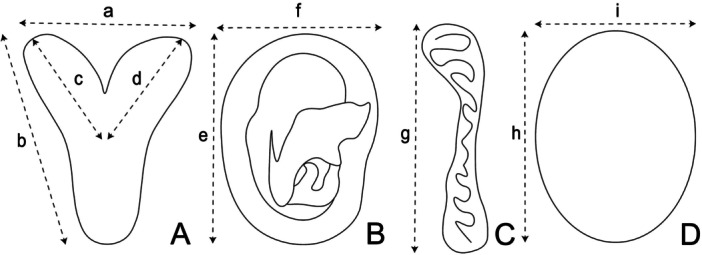
Schematic diagram illustrating the morphometric analysis of the reproductive organs of *Mormoops megalophylla*. (A) Uterine measurements: width (a) and length (b) of the uterus, length of the right uterine horn (c) and length of the left uterine horn (d). (B) Embryonic measurements: width (e) and length (f) of embryos. (C) Epididymal measurements: length (g). (D) Testicular measurements: length (h) and width (i) of testes.

The reproductive tract organs of each specimen, including the testes, epididymides, and uterus, were measured in the laboratory. Measurements were obtained using an American Optical (model 426 10×) ocular micrometer attached to an OLYMPUS stereoscopic microscope (model SZ40). For females, uterine length and width, length of the right and left uterine horns, and dimensions of the embryonic sac were measured (Figure 3A,B). In males, the testicular length and width (Figure 3D) and epididymal length (Figure 3C) were measured according to methodologies described by Danmaigoro et al. (2014) and Cervantes et al. (2008).

The testicular index (TI) was calculated using the following equation:

Testicular index = A/B,where A represents testicular width,and B represents testicular length.

#### 2.3.2 Histological Analysis

Histological staining was conducted to examine structural changes in the ovaries, testicles, and epididymides. The histological procedure followed the protocol described by Montalvo‐Arenas (2010), with tissues stained using hematoxylin (Hycel, Mexico) and eosin (Meyer, Mexico). Observations were performed using a Leica DMLS brightfield microscope (model 4D95), and images were captured utilizing an AmScope digital microscope camera (model MU1000‐HS) and AmScope software version 3.7.

Five images were acquired per field at various magnifications, employing 40×, 100×, 400×, and 1000× objectives. These images have been used to characterize the cellular types involved in spermatogenesis and sperm storage (Beguelini et al. 2014; Bueno et al. 2014). Folliculogenesis has been described by Bueno et al. (2019) and Beguelini et al. (2020).

#### 2.3.3 Classification of Reproductive Stages

The reproductive stages were classified based on morphological, morphometric, and histological variations of the reproductive organs in males and females.

In males, the reproductive cycle stages have been identified through histological evaluation of the testes and epididymides (Danmaigoro et al. 2014; Beguelini et al. 2014; Bueno et al. 2014). Reproductive inactivity was defined as the absence of spermatogenic and/or epididymal activity. Conversely, the reproductive activity stage is characterized by spermatogenic activity in the seminiferous tubules and the presence of spermatozoa in the epididymal ducts.

In females, stages were determined based on variations in the mammary glands, uterus, and ovaries (Sedgeley et al. 2012; Bueno et al. 2019; Beguelini et al. 2020). The onset of reproductive activity was characterized by an increase in uterine dimensions and ovarian follicle development. Gestation stage was defined as a significant increase in uterine size and the presence of an embryo. Lactation was identified by evident mammary gland development; during this stage, nipples become enlarged and protruding, often darkened (keratinized), and are typically surrounded by a bare patch of skin. After nursing, the nipples decrease markedly in size, and the surrounding area becomes covered with fur again Sedgeley et al. 2012). Reproductive inactivity was classified by the absence of characteristics associated with the active reproductive stages.

#### 2.3.4 Statistical Analysis

Statistical analyses considered a significance level of α = 0.05 for both parametric and nonparametric tests. Annual means and confidence intervals were calculated for morphometric variables. Normality was evaluated using the Shapiro–Wilk test. Monthly comparisons were performed separately for each reproductive organ, paired organs (e.g., testes, epididymides, uterine horns), body mass, and BCI. For each organ, monthly means were compared using one‐way ANOVA with Tukey–Kramer post hoc tests when assumptions of normality and homogeneity of variances were met; otherwise, Kruskal–Wallis tests were applied, followed by Dunn's post hoc test.

## 3 Results

### 3.1 Males

#### 3.1.1 Morphology and Anatomy of the Male Reproductive Tract

The male reproductive system of *M. megalophylla* consists of the penis, testicles, associated duct systems (epididymis and vas deferens), and accessory glands (prostate). The bulbourethral glands were not observed during the dissections, and their absence was consistent across all specimens examined throughout the annual cycle. The penis has an average length of 5.68 ± 0.68 mm, featuring a thickened, hair‐covered base that tapers toward the tip, forming a well‐defined glans devoid of hair (Figure 5A). No evidence of a baculum was observed.

The testicles exhibit positional shifts, alternating between the abdominal and scrotal regions throughout the reproductive cycle. They have an average width of 1.63 ± 0.45 mm, length of 2.39 ± 0.60 mm, and TI of 0.68 ± 0.09, all of which vary significantly during the reproductive cycle (width: χ²_(106)_ = 52.86, *p* < 0.001; length: χ²_(106)_ = 45.07, *p* < 0.001; TI: χ²_(106)_ = 45.07, *p* < 0.001; refer to Table 2).

The epididymis averages 3.95 ± 0.75 mm in size and also displays significant reproductive cycle variations (χ²_(108)_ = 33.36, *p* < 0.001; see Table 2).

When comparing the left and right reproductive organs annually, no notable differences were observed in the width and length of the testes or the length of the epididymis (width: *z*_(106)_ = 0.14, *p* = 0.88; length: *t*_(106)_ = 0.30, *p* = 0.76; epididymis: *t*_(108)_ = 0.16, *p* = 0.86). The monthly analyses supported this consistency (see Table 3).

Histological analysis revealed that *M. megalophylla* testicles were paired ovoid structures encased by an external tunica vaginalis and a connective tissue capsule, the tunica albuginea. Each testicle is divided into lobules containing clustered seminiferous tubules surrounded by connective stroma where Leydig cells are located. These seminiferous tubules are covered by their tunica and lined with seminiferous epithelium, which is classified as a complex stratified epithelium (Figure 6).

#### 3.1.2 Reproductive Inactivity Stage

**Figure 4:**
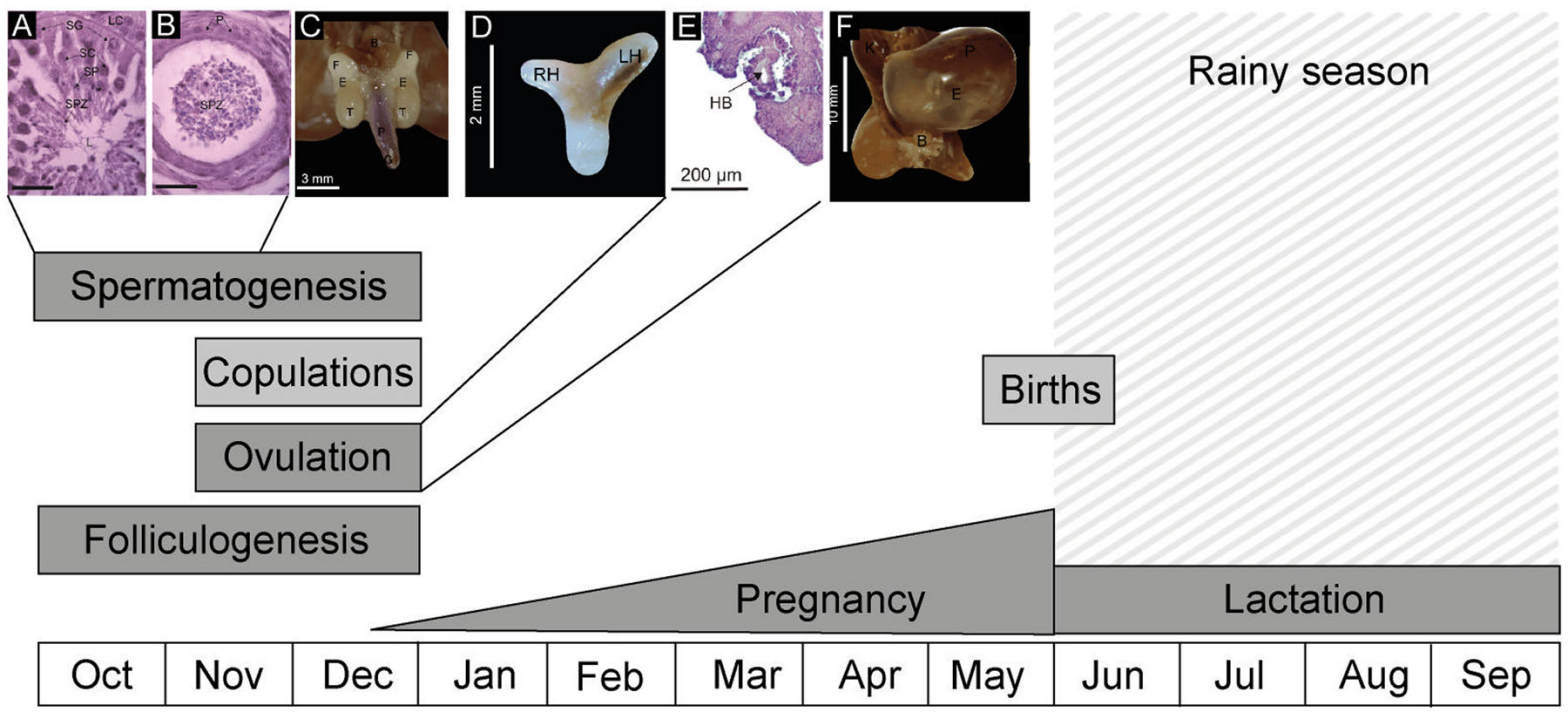
Reproductive cycle of *Mormoops megalophylla* in the cave “El Vado de la Chachalaca,” Carrizal, Veracruz, Mexico. (A) Active seminiferous tubule, (B) epididymis with spermatozoa, (C) male reproductive tract during the period of reproductive activity, (D) uterus at the initiation of gestation, (E) corpus hemorrhagicum (ovulation), and (F) embryo at the terminal stage of gestation. The rainy season is indicated here.

**Figure 5:**
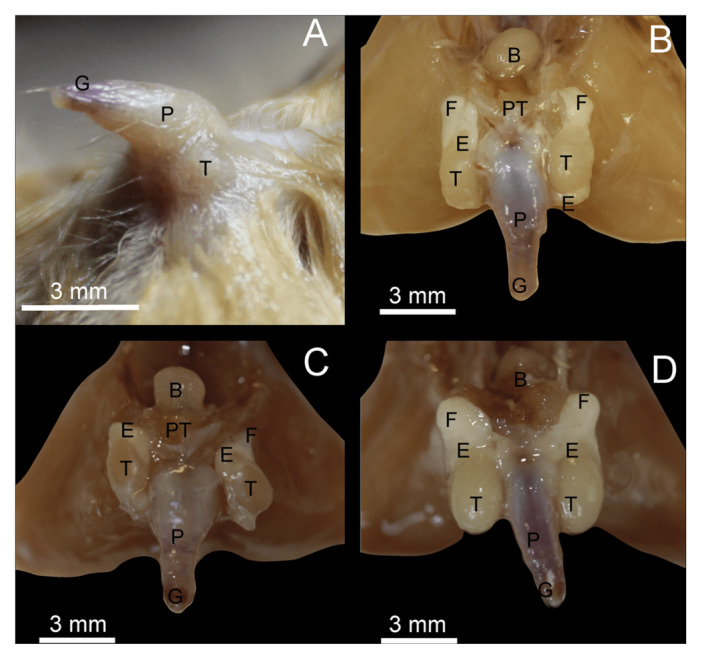
Ventral view of genital organs in the male bat *Mormoops megalophylla*. (A) External anatomy and, following skin removal, during the nonbreeding season in June (B) and February (C), and during the breeding season in October (D). B, bladder; E, epididymis; F, adipose tissue; G, glans; P, penis; PT, prostate; T, testicles.

The reproductive inactivity period of male *M. megalophylla* extends from January to September. The BCI values fluctuated during this phase, with the highest values recorded in July (0.28 ± 0.05) and the lowest in March (0.21 ± 0.01) (Table 2). Testicular position varied between the abdominal and scrotal regions. In most months (e.g., February; Figure 5C), the testes were located abdominally, positioned more cranially and closer to the abdominal cavity. In contrast, in June (Figure 4B), the testes were situated in the scrotal region, more caudally and closer to the base of the penis. The epididymis was not externally visible during this period.

Internally, the testes retained their ovoid shape and were accompanied by well‐defined epididymides that were surrounded by a small layer of fat. Morphometric data indicated that the testicular width (1.22 ± 0.3 mm), testicular length (1.78 ± 0.41 mm), and epididymal length (3.25 ± 0.65 mm) dimensions were smallest in February, whereas the TI reached its lowest level in August (0.58 ± 0.07) (Table 2).

**Figure 6:**
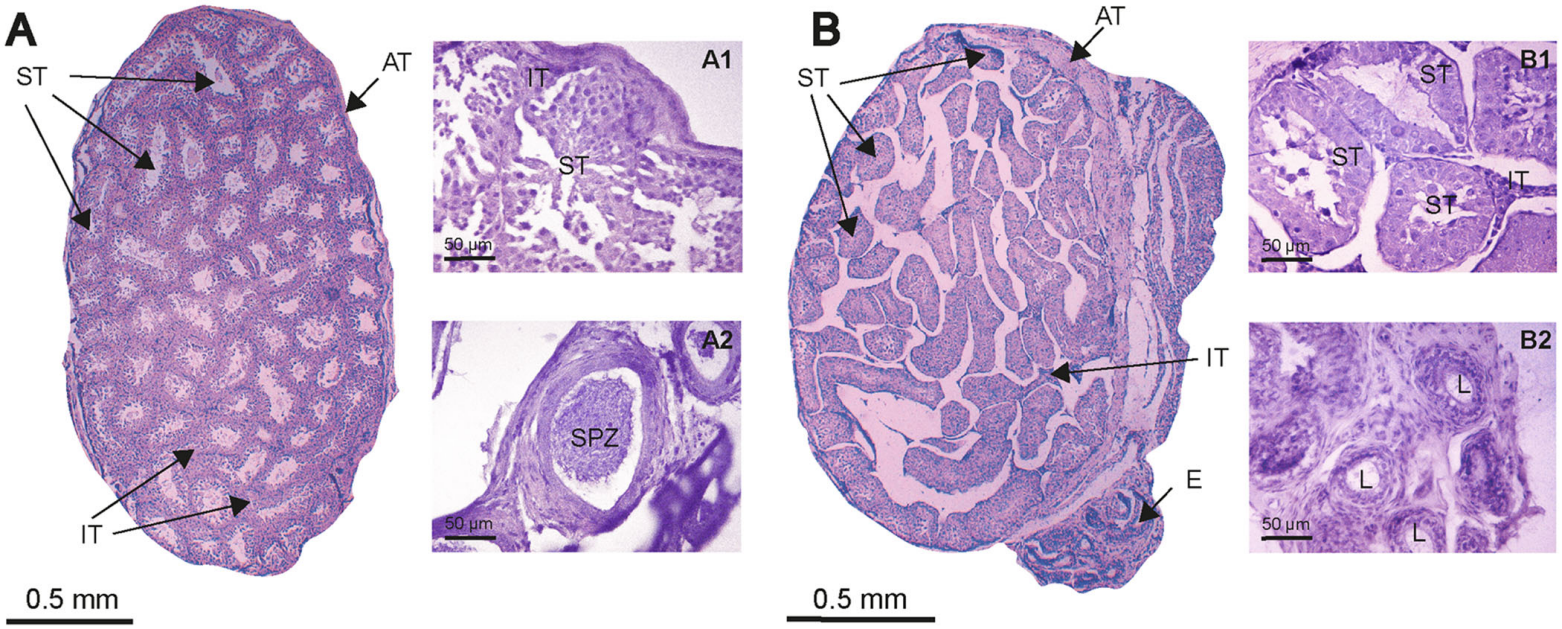
Histological sections of the testis and epididymis of *Mormoops megalophylla* during different reproductive stages. (A) Corresponds to the reproductive activity phase in December; A1 shows active seminiferous tubules, and A2 shows an epididymis with sperm. (B) Corresponds to the reproductive inactivity phase in May; B1 shows inactive seminiferous tubules, and B2 shows an empty epididymis. E, epididymis; IT, interstitial tissue; L, lumen; SPZ, sperm; Staining, hematoxylin–eosin; TA, tunica albuginea; TS, seminiferous tubule.

Histological analysis revealed the absence of spermatogenic activity in seminiferous tubules throughout this phase, as observed in May (Figure 6B). Histological sections revealed inactive seminiferous tubules (Figure 6B1), interstitial tissue, and an epididymis devoid of spermatozoa (Figure 6B2). This condition persisted from February to September.

**Figure 7:**
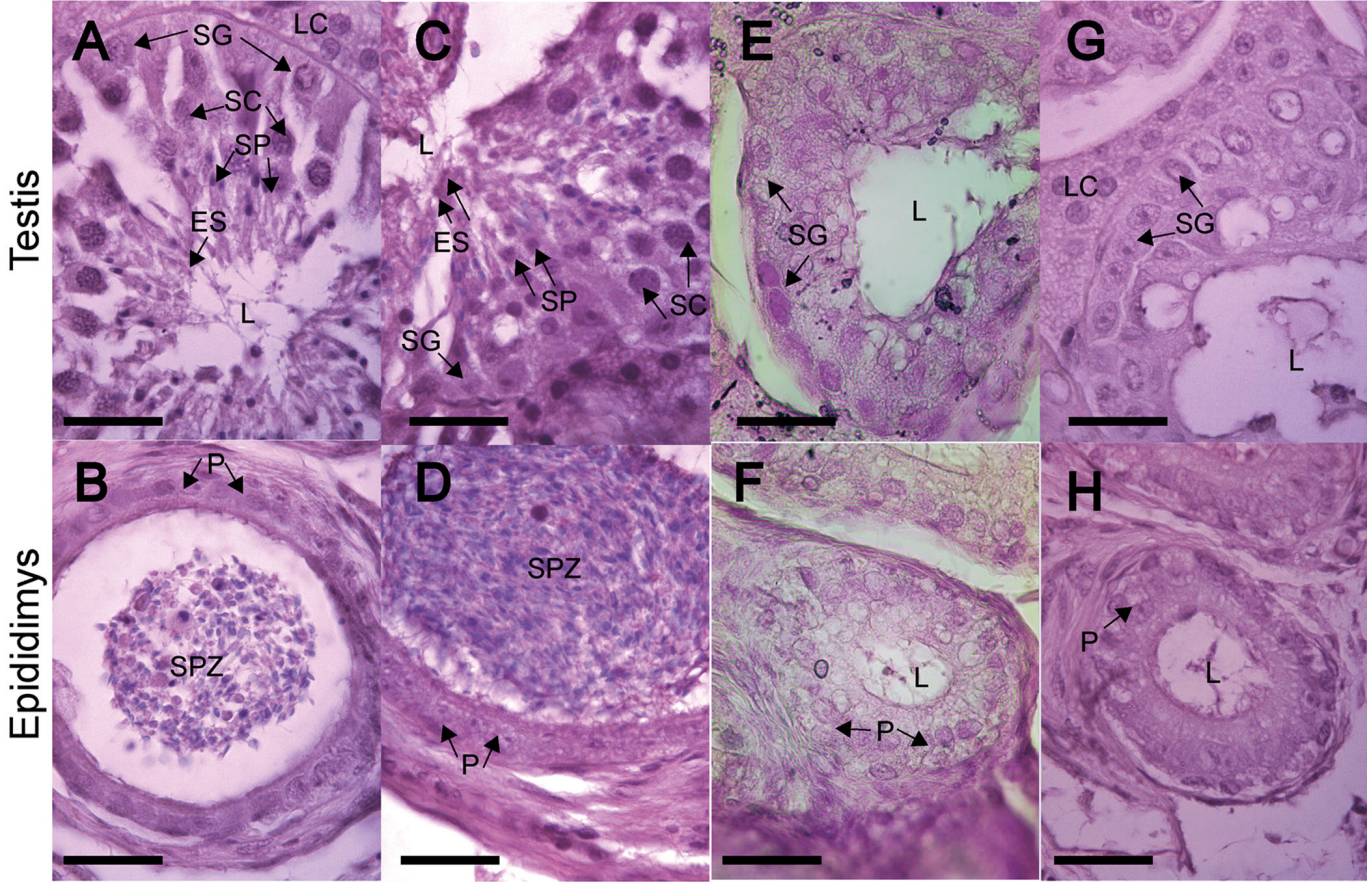
Histological sections of the testis and epididymis of *Mormoops megalophylla* at different months of the reproductive cycle. Active reproductive stage: October (A and B) and December (C and D). Inactive reproductive stage: May (E and F) and June (G and H). ES, elongated spermatids; L, lumen; LC, Leydig cells; P, principal cells; SC, spermatocytes; SG, spermatogonia; SP, spermatids; Staining, hematoxylin–eosin.

During this phase, the seminiferous tubules predominantly contained spermatogonia, with the epididymal lumen entirely empty of spermatozoa, as was observed in May (Figure 7E,F) and June (Figure 7G,H).

#### 3.1.3 Reproductive Activity Stage

Reproductive activity of male *M. megalophylla* occurs between October and December. During this time, the body weight (14.20 ± 0.78 g) and BCI (0.25 ± 0.01) remained constant (Table 2). The testes were exclusively located in the scrotal position and were accompanied by the epididymides (Figure 5D). However, the epididymides were not externally visible before dissection, although they were clearly distinguishable internally. Once exposed, the testes had ovoid shape and were associated with well‐differentiated epididymides surrounded by a greater amount of adipose tissue.

Morphometric data indicated that the highest testicular and epididymal dimensions were recorded in October (testicular width: 2.27 ± 0.21 mm; testicular length: 3.12 ± 0.33 mm; epididymal length: 4.54 ± 1 mm), while the TI reached its peak value in December (0.73 ± 0.05). Histological analyses showed active spermatogenesis in the seminiferous tubules throughout this period (Figure 6 and Figure 6A1,A2) with a well‐organized structure that included interstitial tissue.

Spermatogenesis was evident from the presence of spermatogonia, primary spermatocytes, and elongated spermatids (Figure 7A,C). The lumen of the epididymis was filled with spermatozoa and was surrounded by principal cells (Figure 7B,D). These features remained unchanged throughout the reproductive stage.

The transition to the reproductive inactivity phase commenced in January. Leydig cells, characterized by their large size, polyhedral shape, and lipid inclusions, were observed throughout the annual cycle regardless of spermatogenic status (Figure 7G).

### 3.2 Females

#### 3.2.1 Morphology and Anatomy of the Female Reproductive Tract

The female reproductive system of *M. megalophylla* consists of a pair of ovaries, long bicornuate uterus, and vagina. These reproductive organs are located in the abdominal cavity and surrounded by connective tissue and fat.

**Figure 8:**
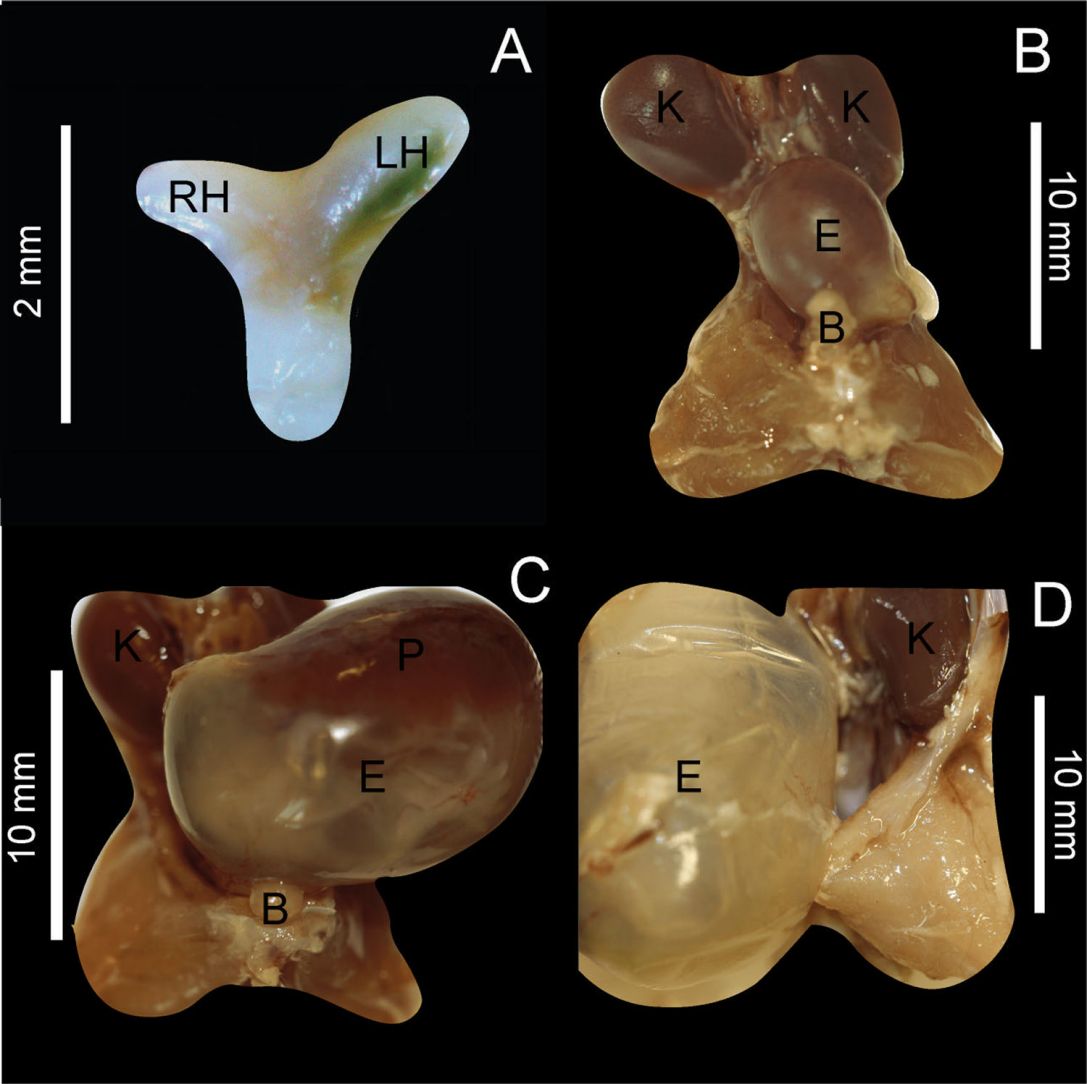
Female reproductive apparatus of *Mormoops megalophylla*. (A) Long bicornuate uterus illustrating its general structure. Pelvic area where the embryo is observed during the gestational period in the months of (B) February, (C) April, and (D) May. B, bladder; E, embryo; K, kidney; LH, left uterine horn; P, placenta; RH, right uterine horn.

The uterus is positioned ventrally within the abdominal cavity, with an average width of 3.00 ± 0.88 mm and a length of 6.59 ± 1.72 mm (Figure 8). Significant morphometric variations in size were observed throughout the reproductive cycle, both in width (*F*_(38)_ = 6.16, *p* = 0.0001) and length (χ²_(38)_ = 23.12, *p* = 0.001), adapting to the presence of an embryo during gestation (Table 2). In contrast, the vagina had an average length of 3.39 ± 0.84 mm and did not exhibit significant variations during the reproductive cycle (χ²_(36)_ = 9.18, *p* = 0.23).

The uterine horns are visible most of the year (Figure 8A) but are more prominent during stages without embryonic presence. During the gestational stages, when the embryo is clearly visible (February–May), the uterine horns are indistinguishable (Figure 8B–D). The left uterine horn has an average length of 1.61 ± 0.59 mm, with significant variations across reproductive stages (χ²_(37)_ = 21.34, *p* = 0.003). In contrast, the right uterine horn, smaller than the left, shows an annual mean length of 1.37 ± 0.36 mm, without significant changes throughout the cycle (*F*_(38)_ = 0.87, *p* = 0.53). Despite these differences in size, no significant differences were found between the average lengths of the uterine horns (*z*(76) = 1.57, *p* = 0.11).

**Figure 9:**
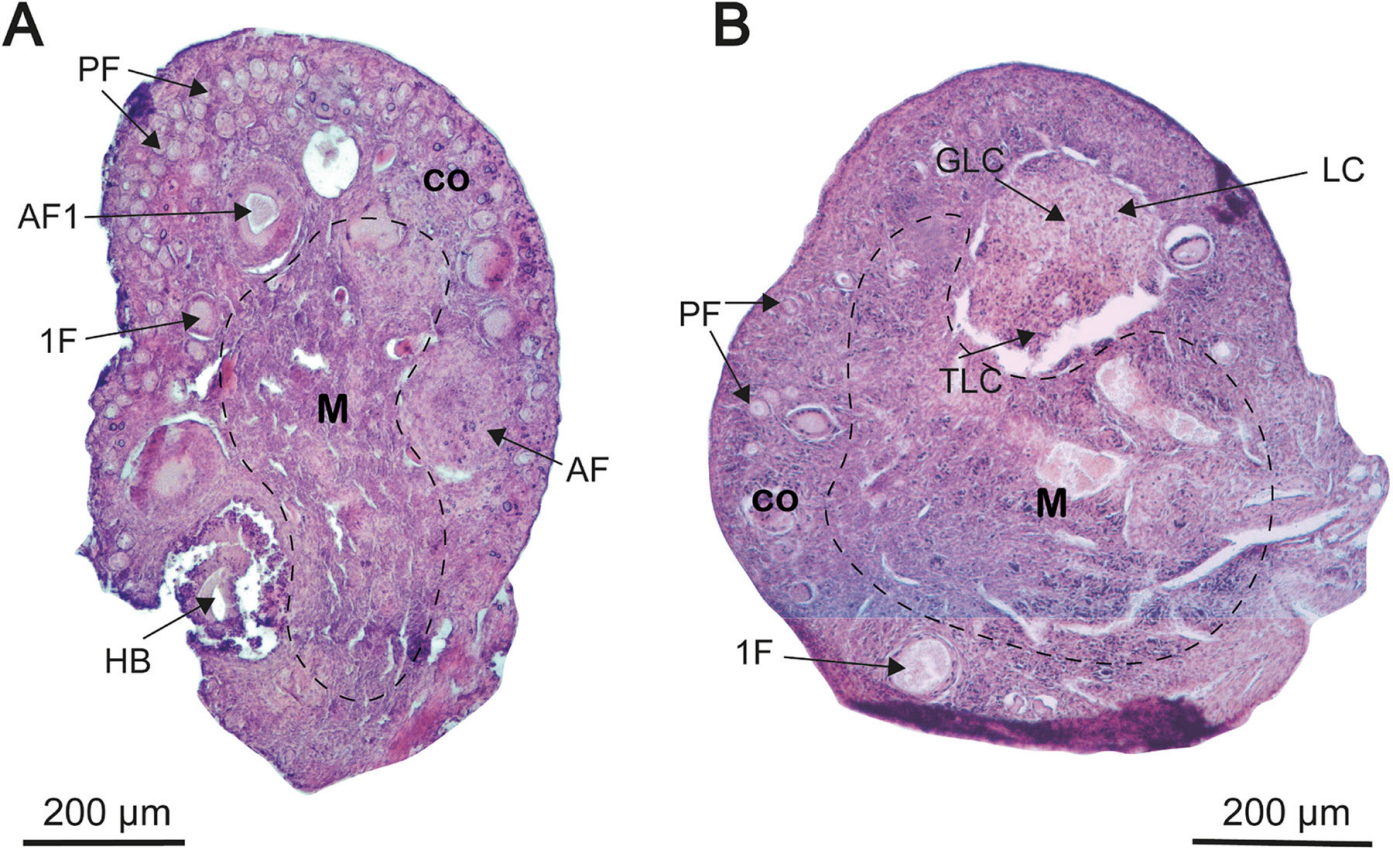
Histological sections of the left ovary of *Mormoops megalophylla* during (A) the onset of reproductive activity and (B) lactation. 1F, primary follicle; AF, follicle in atresia; AF1, advanced primary follicle; BV, blood vessels; GLC, granulosa luteal cells; HB, hemorrhagic body; LC, corpus luteum; PF, primordial follicles; Staining, hematoxylin–eosin; TLC, theca luteal cells.

The ovaries are paired ovoid organs located at the distal end of the uterine horns. They consist of two distinct structures, the cortex and medulla, although no clear boundary separates them (Figure 9). The medulla, which is centrally positioned within the ovary, contains loose connective tissue and blood vessels. The cortex surrounding the medulla is located peripherally and comprises the germinal epithelium (=peritoneal epithelium), ovarian follicles at various developmental stages, and adjacent connective tissue known as stroma. The germinal epithelium is composed of simple cuboidal cells. Additional ovarian structures include the tunica albuginea, which is composed of dense irregular connective tissues and ovarian follicles at different stages of development.

Follicular development varies throughout the reproductive cycle, with dominance of the left ovary over the right ovary. The left ovary is the only ovary that exhibits complete follicular development during the cycle, as detailed in the subsequent images and descriptions.

#### 3.2.2 Beginning of Reproductive Activity

**Figure 10:**
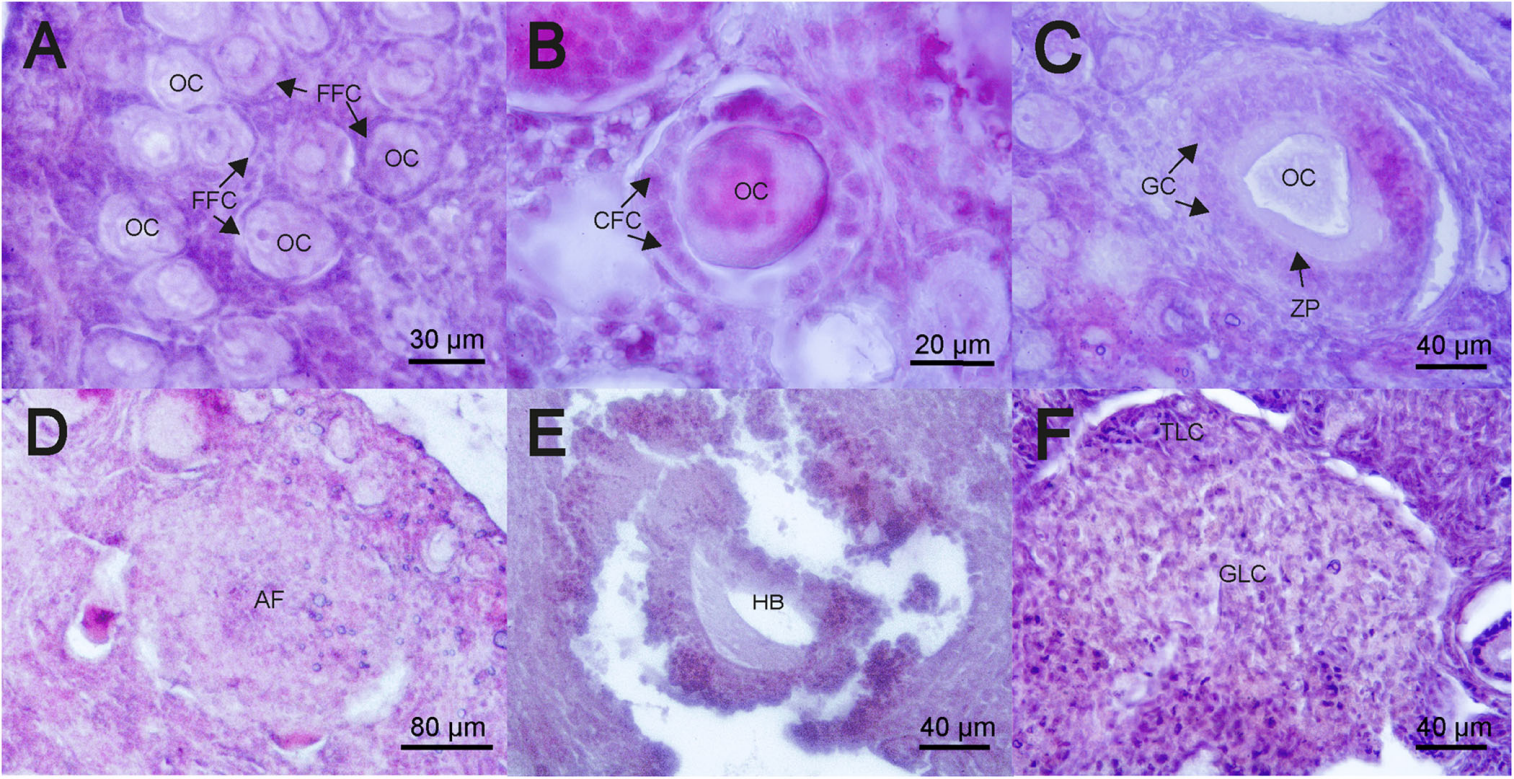
Histological sections of ovarian follicles and luteal bodies in *Mormoops megalophylla*. (A) primordial follicle, (B) primary follicle, (C) advanced primary follicle, (D) atretic follicle, (E) hemorrhagic body, (F) corpus luteum. AF, atretic follicle; CFC, cuboidal follicular cells; FFC, flattened follicular cells; GC, granulosa cells; GLC, granulosa lutein cells; HB, hemorrhagic body; OC, oocyte; Staining, hematoxylin–eosin; TLC, thecal lutein cells; ZP, zona pellucida.

Reproductive activity in *M. megalophylla* females begins in November and December. During this period, body weight (13.5 ± 0.98 g) and BCI (0.24 ± 0.01) remained constant (Table 2). A marked increase in follicular activity was observed, highlighted by the presence of primordial and growing follicles, indicating the initiation of folliculogenesis (Figure 9A). Primordial follicles were located in the cortical stroma beneath the tunica albuginea and consisted of a single layer of flattened follicular cells surrounding an oocyte with a large eccentric nucleus (Figure 10A). Primary follicles were characterized by cuboidal follicular cells (Figure 10B), whereas advanced primary follicles featured stratified granulosa cell epithelium (Figure 10C).

In December (Figure 9A), atretic follicles and follicular ruptures were detected, indicating follicular dominance and ovulation. Follicular rupture was marked by disorganized cellular structures and proximity of atretic follicles to the ovarian cortex, which showed clear evidence of structural disorganization (Figure 10D–E). Moreover, dominance of the left ovary over the right was observed during this period, reflected in the presence of an advanced developmental stage follicle in the left ovary, whereas the right ovary exhibited less significant follicular activity.

#### 3.2.3 Gestational Period

The gestational period of *M. megalophylla* begins in December and is characterized by enlargement of the body and uterine horns. In pregnant females, body weight and BCI gradually increased from February onward, reaching 13.38 ± 0.41 g and 0.24 ± 0.01 g, respectively (Table 2). Notably, the left uterine horn (1.8 ± 0.45 mm) grew more significantly than the right uterine horn (1.29 ± 027 mm) during this period (*U*_(9)_ = 3, *p* = 0.0556). Growth remained steady until January (uterine length: 9.16 ± 0.85 mm, uterine width: 3.79 ± 0.57 mm, left uterine horn: 2.11 ± 0.54 mm, and right uterine horn: 1.47 ± 0.3 mm, Table 2), with the first visible embryos detected in February persisting through May (Figure 8). Embryo size increased over this period, ranging from an average width of 8.37 ± 0.75 mm and length of 6.42 ± 1.20 mm in February to 15.25 ± 0.5 mm in width and 21.75 ± 0.95 mm in length by May (Table 2).

In the initial stages of gestation, particularly in February, embryonic sac structures were not distinctly visible. As gestation progressed, embryonic differentiation became evident in March and April, with the embryo assuming a more defined position. By late May, the embryo had reached its fetal position, accompanied by a well‐defined discoidal placenta (Figure 8C,D).

#### 3.2.4 Lactation Period

**Figure 11:**
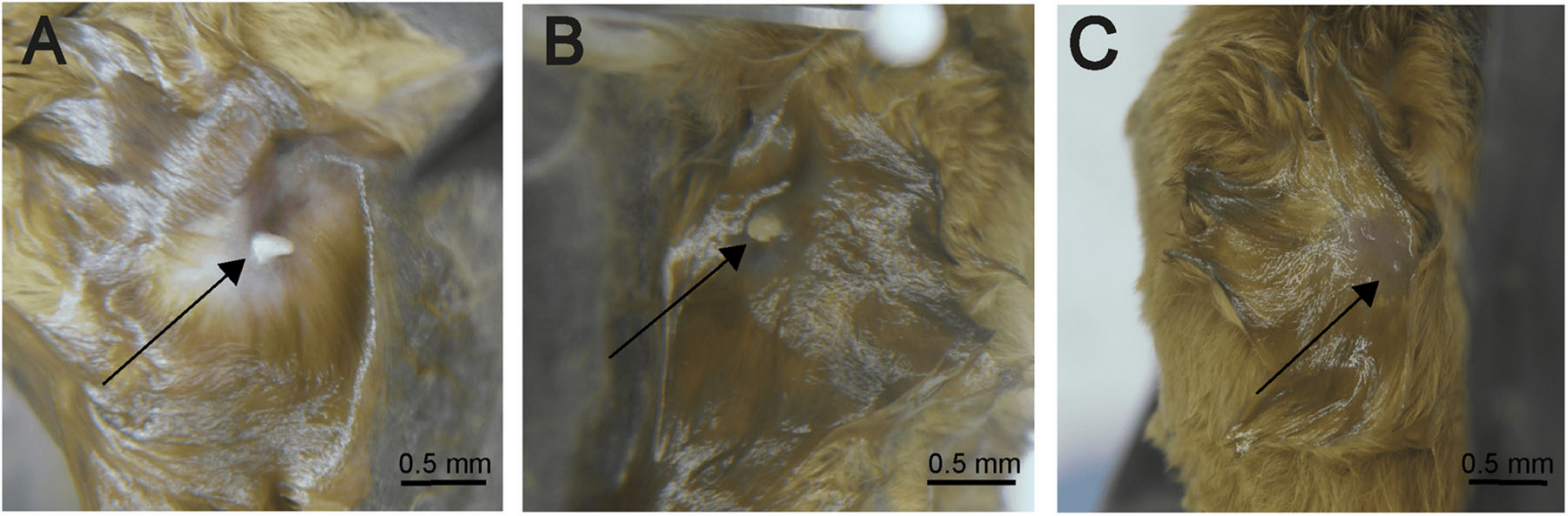
External morphology of the nipple in *Mormoops megalophylla* females during different reproductive stages. (A) Enlarged and protruding nipple during lactation, (B) reduced nipple in the post‐lactation stage, (C) small, inactive nipple outside the reproductive period. The arrows indicate the position of the nipple in each stage.

Lactation was registered between June and September. During this period, a significant reduction in body weight (14.96 ± 0.60 g) and BCI (0.27 ± 0.01) was observed, which persisted throughout the lactation period (Table 2). During lactation, nipples were prominent and surrounded by an area of alopecia (Figure 11A), and these features persisted for up to 4 months. Regarding morphometry, regression of the size of the uterus (uterine length: 6.09 ± 2.06 mm, uterine width: 4.17 ± 1.06 mm) and uterine horns was noted. The left uterine horn (2.32 ± 0.57 mm) remained larger than the right uterine horn (1.65 ± 0.47 mm) during this period (Table 2).

Histologically, in June, the ovaries displayed a corpus luteum (Figures 9B and 10F). These structures were characterized by larger lutein cells from the granulosa, located at the center of the corpus luteum, and smaller, more intensely colored lutein cells from the theca, positioned at the periphery (Figure 10F).

#### 3.2.5 Post‐Lactation and Reproductive Inactivity

*M. megalophylla* females began showing signs of post‐lactation starting in October, a phase during which body weight (13.14 ± 0.75 g) and BCI (0.24 ± 0.01) continued to decrease. At the beginning of this period, the nipples were still prominent but were already covered by fur (Figure 11B). One month later, the nipples became inconspicuous and remained covered with fur, retaining these characteristics until April (Figure 11C).

No significant changes were observed in the morphometry of the uterus during this phase (Table 2). Histologically, the ovaries exhibited primordial follicles and, in some cases, primary follicles. It is important to note that, although these follicles were present, no significant follicular development occurred, suggesting that the females were in a phase of reproductive inactivity.

## 4 Discussion

According to the literature, *M. megalophylla* exhibits a geographically variable reproductive cycle, ranging from a monoestrous pattern with a single reproductive cycle per year to a polyestrous pattern with two or more cycles annually (Table 1). In Mexico, a seasonal monoestrous pattern has been observed in the western (Torres‐Flores et al. 2012) and southern regions (Hernández‐Aguilar and Santos‐Moreno 2020). Populations outside Mexico display polyestrous behavior (Boada et al. 2003).

**Table 1:** Published data on various aspects of the reproductive cycle of *Mormoops megalophylla*. (A) For different locations in Mexico, (B) for different localities outside Mexico.

Reproductive record	Location	Reference
(A)		
Matting season		
March–May	N. Mexico	Villa (1966)
November–January	W. Mexico	Torres‐Flores et al. (2012)
November–December	E. Mexico	This study
Pregnant females		
February–June	S. Mexico	Jones, Smith, et al. (1973); Rezsutek and Cameron (1993)
February–April	S. Mexico	Hernández‐Aguilar and Santos‐Moreno (2020)
March–May	N. Mexico	Villa (1966)
April–July	W. Mexico	Torres‐Flores et al. (2012)
May	W. Mexico	Hernandez et al. (1985)
December–May	E. Mexico	This study
Lactating females		
August	N. Mexico	Wilson et al. (1985)
June–August	S. Mexico	Nowak (1991)
June–September	E. Mexico	This study
Scroted testicles in males		
November–January	W. Mexico	Torres‐Flores et al. (2012)
September, November, December	W. Mexico	Hernandez et al. (1985)
November–February	S. Mexico	Hernández‐Aguilar and Santos‐Moreno (2020)
June, October–December	E. Mexico	This study
(B)		
Reproductive females		
December	Colombia	Arango‐Diago et al. 2020
Matting season		
March–April	Venezuela	Bonaccorso et al. (1992)
Pregnant females		
May and November	Ecuador	Albuja (1999); Boada et al. (2003)
February–June	North America	Beatty (1955); Barbour and Davis (1969); Easterla (1973)
		Jones, Lampe, et al. (1973); Ammerman et al. (2012)
Lactating females		
January, March, October	Ecuador	Boada et al. (2003)
March	Trinidad Isl.	Pavan et al. (2019)
July	Belize	Wynne and Pleytez (2005)
June–September	North America	Yancey (1997)
Scroted testicles in males		
August–September	Colombia	Arango‐Diago et al. 2020

Our findings, which include the presence of spermatozoa in the epididymis, histological evidence of ovulation, progressive uterine enlargement, presence of embryos, and birthing periods, suggest that *M. megalophylla* exhibits a seasonal monoestrous reproductive pattern in the study area. Spermatogenesis and folliculogenesis occur from October to December, the mating season spans November and December, gestation takes place from mid‐December to May, and lactation extends from June to September (Figure 4).

The birthing period and duration of lactation coincided with the rainy season in the region (Figures 2 and 4), aligned with the general reproductive behavior of neotropical bats. This synchronization with environmental conditions ensures the availability of food resources, the abundance of which typically increases during the wet season, thereby enhancing offspring survival (Fleming et al. 1972; Willig 1985; Racey et al. 1987; Heideman 1995; Zortéa 2003).

Our results largely agree with the previously reported stages of the reproductive cycle in Mexico, although with minor temporal differences of 1–2 months (Table 1A). The documented mating season for *M. megalophylla* coincided with reports from western Mexico (Torres‐Flores et al. 2012), which occurred between November and January. In contrast, the proposed gestation period aligns with records from northern, southern, and western Mexico, where pregnant females have been observed between February and July (Villa 1966; Jones, Lampe, et al. 1973; Hernandez et al. 1985; Rezsutek and Cameron 1993; Torres‐Flores et al. 2012).

The observed temporal discrepancies between our findings and those of previous studies can be attributed to two main factors: methodological differences, as prior reports relied exclusively on abdominal palpation to detect pregnancy, and slight variations in environmental conditions, particularly precipitation.

Our findings, along with those from Mexico, Belize (Wynne and Pleytez 2005), and the southern United States (Beatty 1955; Barbour and Davis 1969; Easterla 1973; Jones, Lampe, et al. 1973; Yancey 1997; Ammerman et al. 2012), indicate a consistent reproductive cycle in *M. megalophylla*. Mating occurs from November to December, pregnancy peaks in May and June, and lactation occurs from June to September. This relatively stable temporal pattern suggests that local environmental conditions, such as resource availability, are critical for synchronizing reproductive stages across populations of this species.

In contrast, the Southern Hemisphere populations displayed markedly different reproductive patterns (Table 1B). In Venezuela, mating occurs in March and April (Bonaccorso et al. 1992), representing a shift of 7–8 months compared with our findings. Similarly, pregnant females in Ecuador were observed in May and November (Albuja 1999). Although the May record aligns with the end of the gestation period identified in this study, the November observation is entirely out of sync. Lactating females in Ecuador have been documented in January, March, and October (Boada et al. 2003), whereas in Trinidad Island, lactation was recorded in March (Pavan et al. 2019). These dates did not correspond to our estimated lactation period from June to September.

Given its broad distribution and occurrence in diverse habitats, *M. megalophylla* is subjected to various abiotic factors including temperature, precipitation, photoperiod, and food availability. These factors likely drive the significant differences observed in the reproductive cycles of this species. Reproductive activity in mormoopid bats has been suggested to vary geographically independent of seasonal changes. While data from northern South America support this hypothesis, existing studies often fail to account for environmental factors such as precipitation, a key determinant of reproductive timing in *M. megalophylla* and other neotropical bats, due to increased food availability during rainy periods (Fleming et al. 1972; Willig 1985; Heideman 1995; Zortéa 2003).

As noted by Krutzsch (2000), it is remarkable that the macroscopic structure of the male reproductive tract in *M. megalophylla* has not been previously described. Our observations indicate that it generally follows the pattern typical of most bats, consisting of the penis, paired testes, epididymides, and prostate (Figure 5). The appearance of the penis in *M. megalophylla* is similar in shape and length to that reported for its sister species *Pteronotus mesoamericanus* (Brown et al. 1971). We found no evidence of a penile bone, supporting previous reports of baculum absence in this species and other members of the family Mormoopidae (Brown et al. 1971; Krutzsch 2000). Furthermore, bulbourethral glands were not observed, which could also reflect variation within the family Mormoopidae, since several glands have been reported in *P. mesoamericanus* (Garrido‐Rodríguez et al. 1984). Additionally, no scrotum was observed, which is consistent with the findings in *P. mesoamericanus* (Garrido‐Rodríguez and López‐Forment 1981; Garrido‐Rodríguez et al. 1984), as well as in other bat species, such as *Taphozous hilli* (Kitchener 1980), *Mormopterus planiceps* (Crichton and Krutzsch 1987), and *Tadarida brasiliensis* (Krutzsch 1955). The epididymides, as in other mammals, can be divided into typical head, body, and tail regions, although none of these structures are externally visible.

Interestingly, we did not detect several accessory glands previously reported in other bats, including those described for two other species within the same family. We identified only the prostate, aligning with descriptions across all bat families studied to date (Krutzsch 2000). However, contrasting evidence exists for *Pteronotus gymnonotus*, where urethral glands, a pair of bulbourethral and ampullary glands, and the absence of seminal vesicles have been documented (Pimentel et al. 2023). Similarly, *P. mesoamericanus* has been shown to possess a prostate, seminal vesicle, ampullary, urethral, and Cowper's glands, with accessory gland hypertrophy occurring during the mating season (Garrido‐Rodríguez et al. 1984).

In our study, no such glands were identified in reproductively active or inactive specimens of *M. megalophylla* (Figure 5). However, it is possible that the glands are present but too small to be detected macroscopically. Therefore, more detailed microscopic and histological analyses are required to confirm their absence in this species.

The male reproductive system exhibits morphological and functional variations throughout the reproductive cycle, as reflected by changes in size and activity. Seasonal testicular migration was detected from the abdominal to the inguinal region (Figure 5B,D). During the reproductive period, the testes significantly increased in size and adopted a parapenile position (Figure 5D), a feature that has likely been misinterpreted as scrotal testes in previous studies (Hernandez et al. 1985; Torres‐Flores et al. 2012; Hernández‐Aguilar and Santos‐Moreno 2020).

Seasonal testicular migration is considered a mechanism that ensures optimal temperature for sperm storage and maintenance in the epididymis (Jolly and Blackshaw 1988). Morphometric analyses revealed an increase in testicular size and TI during the reproductive activity phase (Table 2), a phenomenon not previously described for *M. megalophylla*. These increases serve as critical indicators of gonadal activity, consistent with observations in other mormoopid bats, such as *Pteronotus mesoamericanus*, where testicular size varies across the reproductive cycle (Garrido‐Rodríguez et al. 1984). Testicular enlargement in bats has been linked to increased spermatogenic activity, reflecting significant energetic investment in reproductive tissues (Kenagy and Trombulak 1986; Beguelini et al. 2014).

**Table 2:** Mean monthly values ± standard deviation of body mass, body condition index (BCI), and morphometric variables of the reproductive structures of male and female *Mormoops megalophylla* obtained during the present study. *p* value, *F*‐value (or *H* ^a^), and degrees of freedom (df) are presented for each comparison.

Female															
	Pregnancy		Lactation				Post‐lactation	Beginnin of reproductive activity		Pregnancy			*p*	*F* (or *H* a)	gl
			Folicular inactivity					Pregnancy							
	Apr	May	Jun	Jul	Aug	Sep	Oct	Nov	Dec	Jan	Feb	Mar			
Body weight (g)	15.16 ± 1.11	15.06 ± 1.79	14.96 ± 0.60	14.62 ± 1.69	14.46 ± 1.44	13.14 ± 0.75	13.14 ± 1.00	13.02 ± 0.93	13.98 ± 0.85	13.08 ± 0.75	13.38 ± 0.41	14.7 ± 0.42	0.01	22.25^a^	54
BCI	0.3 ± 0.06	0.27 ± 0.04	0.27 ± 0.01	0.26 ± 0.03	0.27 ± 0.02	0.23 ± 0.01	0.24 ± 0.01	0.24 ± 0.02	0.25 ± 0.01	0.24 ± 0.01	0.24 ± 0.01	0.27 ± 0.01	0.002	27.13^a^	54
Uterine length (mm)	—	—	6.09 ± 2.06	5.74 ± 0.52	5.46 ± 0.49	5.23 ± 0.46	6.33 ± 0.77	5.98 ± 1.37	8.73 ± 0.92	9.16 ± 0.85	—	—	< 0.001	6.16	38
Uterine width (mm)	—	—	4.17 ± 1.06	2.7 ± 0.24	2.43 ± 0.08	2.99 ± 0.90	2.34 ± 0.55	2.21 ± 0.35	3.27 ± 0.60	3.79 ± 0.57	—	—	0.001	23.12^a^	38
Left uterine horn (mm)	—	—	2.32 ± 0.57	1.19 ± 0.21	1.06 ± 0.60	1.66 ± 0.36	1.11 ± 0.23	1.38 ± 0.51	1.8 ± 0.45	2.11 ± 0.54	—	—	0.003	21.34^a^	37
Right uterine horn (mm)	—	—	1.65 ± 0.47	1.46 ± 0.30	1.14 ± 0.16	1.26 ± 0.51	1.32 ± 0.27	1.31 ± 0.41	1.29 ± 0.27	1.47 ± 0.30	—	—	0.53	0.87	38
Embryonic width (mm)	14.2 ± 1.92	15.25 ± 0.50	—	—	—	—	—	—	—	—	8.37 ± 0.75	10 ± 1.41	0.01	8.07^a^	20
Embryonic length (mm)	20.6 ± 1.14	21.75 ± 0.95	—	—	—	—	—	—	—	—	6.42 ± 1.20	14.5 ± 2.12	< 0.001	243.90	14

Male															
	Reproductive inactivity						Reproductive activity			Reproductive inactivity			*p*	***F*** **(or** ***H*** a **)**	**gl**
	Apr	May	Jun	Jul	Aug	Sep	Oct	Nov	Dec	Jan	Feb	Mar			
Body weight (g)	13.94 ± 0.83	13.4 ± 1.14	14.52 ± 0.88	15.78 ± 2.36	14.16 ± 1.05	13.2 ± 0.62	14.32 ± 0.58	14.44 ± 1.02	13.86 ± 0.74	14.08 ± 1.03	12.72 ± 1.54	11.78 ± 0.78	0.008	25.31^a^	59
BCI	0.25 ± 0.01	0.24 ± 0.02	0.26 ± 0.02	0.28 ± 0.05	0.26 ± 0.02	0.24 ± 0.01	0.26 ± 0.01	0.26 ± 0.02	0.25 ± 0.01	0.25 ± 0.01	0.23 ± 0.03	0.21 ± 0.01	0.006	25.99^a^	59
Testicular width (mm)	1.54 ± 0.27	1.55 ± 0.21	1.59 ± 0.21	1.5 ± 0.20	1.31 ± 0.32	1.35 ± 0.39	2.27 ± 0.21	2.04 ± 0.51	2.08 ± 0.17	1.66 ± 0.52	1.22 ± 0.30	1.43 ± 0.25	< 0.001	53.56^a^	105
Testicular length (mm)	2.32 ± 0.35	2.12 ± 0.22	2.32 ± 0.28	2.39 ± 0.23	2.28 ± 0.67	2.02 ± 0.71	3.12 ± 0.33	3.04 ± 0.74	2.87 ± 0.33	2.3 ± 0.68	1.78 ± 0.41	2.24 ± 0.36	< 0.001	45.07^a^	106
Testicular index	0.67 ± 0.11	0.73 ± 0.10	0.7 ± 0.12	0.62 ± 0.07	0.58 ± 0.07	0.68 ± 0.07	0.72 ± 0.03	0.67 ± 0.08	0.73 ± 0.05	0.72 ± 0.08	0.68 ± 0.08	0.64 ± 0.07	< 0.001	45.11^a^	105
Epididimal length (mm)	3.97 ± 0.42	3.85 ± 0.28	4.41 ± 0.42	3.9 ± 0.52	4.04 ± 0.63	3.33 ± 0.76	4.54 ± 1	4.53 ± 1.04	4.28 ± 0.44	3.8 ± 0.62	3.25 ± 0.65	3.58 ± 0.60	< 0.001	33.36^a^	108

^a^ A non‐parametric statistic (Kruskal–Wallis test).

From January to September, males exhibited abdominal testes, whereas from October to December, the testes were more developed and exclusively in the inguinal position across all captured males. Interestingly, in June, all the males had larger inguinal testes. These findings suggest the presence of two peaks in the inguinal testicular position, similar to the bimodal seasonal polyestry observed in *Molossus molossus* (Soares et al. 2020). However, in *M. megalophylla*, the June peak occurred during reproductive inactivity, suggesting that testicular positioning is not exclusively linked to spermatogenic activity.

One possible explanation for testicular enlargement and descent during reproductive inactivity is their association with thermoregulation, which is essential for maintaining testicular functionality over time, even in the absence of spermatogenesis. Testes may require specific temperatures to preserve the structural and functional integrity of their tissues (Jolly and Blackshaw 1988; Viana et al. 2022).

No significant morphological differences were found between the left and right testes in width, length, and TI (Table 3), nor in the lengths of the epididymides on either side. These findings suggest symmetrical development of these organs, which has not been previously reported for the family Mormoopidae or for this species. This observation supports the hypothesis of functional symmetry in both testes and epididymides.

**Table 3:** Monthly comparison of morphometric measurements of left and right testes and epididymal lengths of *Mormoops megalophylla* during the present study. *p* value, *t*‐value (or *z* ^a^), and degrees of freedom (df) are presented for each comparison.

Month	Testicle width		*p*	*t* (or *z* a)	gl	Testicle length		*p*	*t* (or *z* a)	gl	Epididymal length		*p*	*t* (or *z* a)	gl
	Left	Right				Left	Right				Left	Right			
April	1.55 ± 0.27	1.53 ± 0.31	0.93	0.08	7	2.38 ± 0.36	2.26 ± 0.37	0.65	0.46	7	4.12 ± 0.35	3.28 ± 0.47	0.33	1.03	7
May	1.51 ± 0.20	1.58 ± 0.24	0.68	0.42	7	2.11 ± 0.23	2.13 ± 0.24	0.92	0.10	7	3.85 ± 0.19	3.85 ± 0.39	0.99	0.005	7
June	1.63 ± 0.19	1.74 ± 0.58	0.40	0.83^a^	9	2.19 ± 0.23	2.46 ± 0.29	0.14	1.50	9	4.30 ± 0.47	4.51 ± 0.39	0.46	0.76	9
July	1.53 ± 0.17	1.46 ± 0.23	0.67	0.43	7	2.39 ± 0.16	2.40 ± 0.31	0.95	0.06	7	3.86 ± 0.16	3.99 ± 0.78	0.87	0.16	7
August	1.28 ± 0.29	1.35 ± 0.39	0.75	0.32	8	2.22 ± 0.67	2.36 ± 0.75	0.78	0.28	8	3.83 ± 1.63	4.29 ± 0.60	0.31	1.08	8
September	1.28 ± 0.30	1.45 ± 0.50	0.54	0.61^a^	8	1.90 ± 0.61	2.17 ± 0.89	0.53	0.61^a^	8	3.12 ± 0.70	3.54 ± 0.84	0.14	1.46^a^	9
October	2.35 ± 0.17	2.19 ± 0.24	0.33	1.04	7	3.21 ± 0.25	3.03 ± 0.41	0.49	0.72	7	4.47 ± 1.22	4.67 ± 0.79	0.75	0.31	8
November	2.08 ± 0.50	1.98 ± 0.59	0.54	0.61^a^	8	3.07 ± 0.71	3.01 ± 0.89	0.91	0.11	8	4.73 ± 1.11	4.38 ± 1.08	0.64	0.47	8
December	2.18 ± 0.17	1.99 ± 0.11	0.07	2.07	9	3.02 ± 0.42	2.72 ± 0.14	0.14	1.46^a^	9	4.32 ± 0.30	4.25 ± 0.58	0.79	0.27	9
January	1.75 ± 0.59	1.56 ± 0.51	0.63	0.49	7	2.27 ± 0.75	2.34 ± 0.71	0.89	0.13	7	3.92 ± 0.66	3.69 ± 0.66	0.66	0.43^a^	7
February	1.17 ± 0.35	1.27 ± 0.26	0.33	0.95^a^	9	1.73 ± 0.33	1.86 ± 0.50	0.60	0.54	9	3.31 ± 0.81	3.19 ± 0.53	0.78	0.28	9
March	1.46 ± 0.97	1.39 ± 0.27	0.68	0.41	9	2.16 ± 0.39	2.32 ± 0.36	0.54	0.63	9	3.61 ± 0.61	3.55 ± 0.66	0.88	0.14	9

^a^ A non‐parametric statistic (Dunn's post hoc test).

Histological analyses of the seminiferous tubules revealed typical mammalian cellular types (Tortora and Derrickson 2006; Ross and Pawlina 2016), with distributions and variations consistent with reproductive stages (Figures 6 and 7). Based on the classification by Bueno et al. (2014), the male reproductive cycle in *M. megalophylla* can be divided into four phases: the active phase (October–December), regression phase (January), regressive phase (from February onward), and recrudescence phase (late September–early October).

During the active phase, constant spermatogenic activity was observed, with testes in a scrotal position and larger in size than those in the inactive phase. This synchronized pattern across the studied individuals contrasts with the asynchronous spermatogenesis reported in *Pteronotus mesoamericanus* by Garrido‐Rodríguez et al. (1984). Such differences may be attributed to species‐specific variations in reproductive physiology and mechanisms regulating spermatogenic synchronization. These variations may be influenced by environmental conditions, which play a key role in shaping the reproductive seasonality of each species.

In the epididymis, principal cells, which are essential for sperm maturation and epididymosome formation (Robaire et al. 2006; Sullivan and Mieusset 2016), predominate throughout the annual cycle. Variations were limited to the presence or absence of spermatozoa in the tubular lumen, which was synchronized with testicular activity, reflecting functional coordination during reproductive stages. These findings are consistent with those reported for *Pteronotus mesoamericanus* (Garrido‐Rodríguez et al. 1984), ruling out prolonged sperm storage in mormoopid bats.

The histomorphometric approach enabled a more precise description of gonadal variations in the studied species, allowing a detailed characterization of changes associated with the reproductive cycle. Compared to previous studies that relied solely on visual determination of testicular position, this integrative approach highlights the importance of using more precise methodologies and extended temporal monitoring to better understand reproductive stages in neotropical bats.

The female reproductive system of *M. megalophylla* retains the basic structure of eutherian mammals and is composed of the vagina, cervix, uterus, two oviducts, and two ovaries. This anatomy is similar to that described for other species of the Mormoopidae family (Hood and Smith 1983), such as *Pteronotus gymnonotus* (Costa and Morais 2022) and *P. mesoamericanus* (Garrido‐Rodríguez et al. 1984). All of these species exhibit a bicornuate uterus with short uterine horns and a common uterine body characterized by a prominent internal cavity (Figure 8).

A distinctive feature of *M. megalophylla* is the marked asymmetry of the uterine horns; the left horn is anatomically and functionally hypertrophic. This pattern contrasts with what has been reported for *Pteronotus davyi*, *P. rubiginosa*, and *P. parnellii* (Wimsatt 1979; Hood and Smith 1983), suggesting that hypertrophy of the uterine horns may vary among species within this family.

No prior studies have described the characteristics of the vagina. However, our results indicate that its length does not show significant variations during the reproductive cycle, suggesting that unlike the uterus, this organ does not undergo morphometric changes related to the analyzed reproductive stages.

Throughout the reproductive cycle, significant variations were detected in uterus size, both in width and length. These changes were directly associated with the presence of the embryo during gestation, reflecting the physiological adaptations of the female reproductive system across different reproductive stages. These findings indicate hormonal regulation that modulates uterine growth during gestation (Ross and Pawlina 2016).

No significant differences in size or shape were observed between the ovaries of *M. megalophylla*. However, only the left ovary exhibited typical follicular development throughout the annual cycle, while the right ovary consistently contained only primordial and primary follicles. Follicular development occurred exclusively in the left ovary, which is consistent with the left dominance of the uterine horn. This phenomenon has also been documented in other bat species (Wimsatt 1979; Pillai and Sastry 2012), as well as in *Artibeus planirostris* (Bueno et al. 2019) and in species of the family Vespertilionidae, including *Miniopterus schreibersii*, *Miniopterus australis*, and *Miniopterus fraterculus* (Richardson 1977; Bernard 1980).

This left ovarian dominance throughout the reproductive cycle, with complete follicular development observed at various stages. The asymmetry observed in our results was characterized by complete left‐sided dominance, where the left ovary and uterine horn were functionally dominant. These findings align with previous suggestions regarding the functional capacity of the female reproductive tract in bats (Rasweiler and Badwaik 2000; Pillai and Sastry 2012).

In contrast, these findings differed from those reported for other Mormoopidae species. For example, functional activity has been recorded in both ovaries of *Pteronotus gymnonotus* (Costa and Morais 2022), whereas morphological and functional asymmetry favoring the right side has been reported in *P. parnellii* and *P. mesoamericanus* (Badwaik and Rasweiler 1998; Garrido‐Rodríguez et al. 1984). In addition, right uterine horn dominance has been documented in *Pteronotus davyi*, *P. rubiginosa*, and *P. parnellii* (Hood and Smith 1983; Badwaik and Rasweiler 1998). These differences might reflect the diversity of reproductive strategies within the Mormoopidae family.

In a specific case of *M. megalophylla*, left‐sided ovarian dominance could result from successive alternating ovulations favoring the left uterine horn, differential stimulation of the oviducts, or uterine horns. It is important to note that studies on this family remain scarce and the sample sizes in these studies were generally small.

The reproductive activity of the female *M. megalophylla* begins between November and December. During this period, body weight and BCI remained stable, suggesting that energy investment related to reproduction is not yet substantial in this early phase of the cycle (Hayssen et al. 1993).

At the beginning of December, primordial and growing follicles were observed, clearly indicating that females were preparing for ovulation (Figure 9A). In addition, the presence of atretic follicles and follicular rupture signals the dynamic process of follicular dominance and ovulation (Bueno et al. 2019; Ferraz et al. 2023). Although there are no previous records of follicular dynamics in *M. megalophylla*, other studied mormoopids, such as *P. gymnonotus*, do not exhibit a marked seasonal pattern in follicular dynamics, as primary, secondary, Graafian, and atretic follicles are present at any time of the year (Costa and Morais 2022).

Gestation in *M. megalophylla* begins in December, coinciding with ovulatory follicular activity, and extends until May. During this period, from February onward, in the mid‐gestation phase, a gradual increase in body weight and BCI was observed, which is attributed to embryonic growth. This increase is also indicative of energy accumulation in pregnant females, which is crucial to support the high metabolic costs associated with gestation and subsequent lactation (McLean and Speakman 2000).

Our results showed a corpus luteum with histological characteristics similar to those described in other bat species (Bueno et al. 2019; Rodrigues et al. 2019; Beguelini et al. 2020). However, unlike most mammals, we observed the persistence of the corpus luteum until the onset of lactation, suggesting that in *M. megalophylla*, the corpus luteum persists longer than in other species. This feature is typical of seasonal monestrous species, in contrast to the short‐lived corpus luteum (present only during early gestation) observed in polyestrous species (Williams and Erickson 2000; Stouffer et al. 2013). This phenomenon has also been reported in other bat species, where the corpus luteum remains partially organized until the end of pregnancy and the onset of lactation (Beguelini et al. 2020). Once formed, the placenta plays a role in progesterone production, inducing luteolysis of the corpus luteum (Dale Buchanan and Younglai 1986; Gopalakrishna et al. 1986; Van Aarde et al. 1994; Towers and Martin 1995). However, our findings suggest that persistence of the corpus luteum plays a crucial role in sustained progesterone production throughout gestation, highlighting an adaptive reproductive strategy that ensures the necessary hormonal support until birth and the initiation of lactation.

We observed that *M. megalophylla* exhibits a well‐defined discoidal chorioallantoic placenta at the end of gestation, a placental type that is widespread among bats (Crichton and Krutzsch 2000). The macroscopic placental morphology is consistent with that described in other bat species, including *Myzopoda aurita* y *M. schliemanni* (Carter et al. 2008), *Myotis albescens* (Rodríguez et al. 2018), and *P. parnellii* (Badwaik and Rasweiler 1998). This structure facilitates efficient nutrient and gas exchange between the mother and embryo during later stages of development (Roa et al. 2012).

Uterine growth, which is an indicator of embryonic development, continues steadily throughout gestation. By February, the embryonic sac was clearly differentiated, although with poorly defined structures, and continued to develop until the embryo reached a clearly visible fetal position in April and May. The embryo size increased during gestation, with a crown‐rump length of 21.75 ± 0.95 mm by May (Table 2), which is smaller than that recorded for *P. parnellii* (23.0–25.0 mm—Badwaik and Rasweiler 1998) and *P. mesoamericanus* (27–28 mm—Garrido‐Rodríguez et al. 1984).

In the study area, the gestation period of *M. megalophylla* lasted approximately 6 months, beginning in December and concluding in May. Torres‐Flores et al. (2012) reported a gestation duration of 4 months, but this estimate is based on abdominal palpation detection, which hinders the accurate identification of early gestation stages and introduces a bias in detecting the gestational process. In contrast, our assessment, based on anatomical analyses, provides a more precise gestational chronology. This methodological rigor is consistent with the approach used in *Carollia perspicillata*, where gestation length was determined by dissection (Cretekos et al. 2005).

The lactation period in *M. megalophylla*, extending from June to September, was accompanied by a sustained decrease in body weight and BCI. This decline is consistent with observations in other bat species, where the energetic investment required for milk production and offspring care directly affects the mother's physical condition, reflecting the high metabolic costs associated with lactation, a highly energy‐demanding process for females (Elangovan et al. 2010).

The lactation period previously reported for this species by Wilson et al. (1985), and Nowak (1991) fully aligns with our results (Table 1), indicating that conspicuous nipples, surrounded by alopecia, serve as a reliable indicator of this reproductive stage in the species. The characteristics of nipples during lactation function as an adaptation that facilitates contact with the offspring, thereby improving the efficiency of milk transfer (Kunz et al. 2010).

Our histological analysis showed that during lactation, there was regression in the size of the uterus and uterine horns toward their basal state, indicating that gestation had been concluded. This observation has also been documented in other neotropical bat species, such as *Artibeus lituratus* (Rodrigues et al. 2019).

The histomorphometric analysis approach allowed for a more precise description of gonadal variations in the species under study, providing detailed insights into the changes associated with the reproductive cycle. Compared to previous studies that focused solely on the scrotal position, this comprehensive approach emphasizes the importance of more precise methodologies and a broader temporal follow‐up, enabling a deeper understanding of reproductive stages in tropical bats (Krutzsch 2000; Racey 2009).

It is essential to emphasize that the study of the reproductive cycle of *M. megalophylla* in a tropical dry forest in western Mexico reveals a synchronous seasonal monoestrous pattern, likely widespread throughout the species' distribution from Central America to the northern United States. Copulation occurs between November and December, gestation extends from December to late May, and births occur in June, coinciding with the rainy season. Ovulation occurs in the left ovary, and implantation and embryonic development are restricted to the left uterine horn, indicating functional asymmetry favoring the left side. Furthermore, the left ovary showed a long‐lasting corpus luteum, which persisted even after parturition.

Males exhibit reproductive synchronization with females. Their testes are migratory, with two inguinal periods: the first is in June, when spermatogenesis is absent, and the second is from October to December, which coincides with the sole period of male reproductive activity. This period is characterized by a 3‐month spermatogenic cycle (October to December), during which spermatozoa are present in the epididymis and ready for copulation.

Finally, although the methodologies employed in this study have been crucial for describing the stages of the reproductive cycle, it is important to note that studies on hormonal control in bats remain limited (Beguelini et al. 2021). Annual hormonal fluctuations are closely linked to reproductive variation (Krishna and Bhatnagar 2011). However, no detailed studies have been conducted on the hormonal concentrations associated with the reproductive patterns of *M. megalophylla* and other species of the family Mormoopidae.

Future research should focus on characterizing hormonal variations in *M. megalophylla* and linking them to the reproductive events described in this study. In addition to hormone concentration analyses, immunohistochemical studies targeting key markers of the female and male reproductive cycles would provide further insights into the cellular and molecular mechanisms involved. Such approaches would significantly enhance our understanding of reproductive physiology and contribute valuable information on the endocrine regulation of bat reproduction. This study not only broadens our knowledge of tropical bat biology but also establishes a foundation for comparative studies across species, aiding in the advancement of their ecological and conservation strategies.

## Author Contributions

**Gihovani Ademir Samano‐Barbosa:** investigation, methodology, conceptualization, writing – original draft, writing – review and editing, formal analysis. **Ixchel Rojas‐Martínez:** methodology, investigation. **Sergio Leonardo Porto‐Ramírez:** investigation, methodology. **Fernando Salgado‐Mejia:** investigation, methodology, writing – original draft, writing – review and editing, formal analysis. **Ahiezer Rodríguez‐Tobón:** formal analysis. **Arturo Salame‐Méndez:** formal analysis. **Edith Arenas‐Ríos:** formal analysis. **Luis Manuel Guevara‐Chumacero:** funding acquisition, investigation, formal analysis, methodology. **Ricardo López‐Wilchis:** investigation, funding acquisition, formal analysis, supervision, resources, project administration, writing – review and editing, writing – original draft, methodology, conceptualization.

## Peer Review

The peer review history for this article is available at https://www.webofscience.com/api/gateway/wos/peer-review/10.1002/jmor.70066.

## Data Availability

The data that support the findings of this study are available on request from the corresponding author. The data are not publicly available due to privacy or ethical restrictions.
